# Strengthening Saudi Arabia’s Primary Health Care through an e-Referral System: A Case Study

**DOI:** 10.3390/clinpract12030042

**Published:** 2022-05-24

**Authors:** Khalid H. Alabbasi, Estie Kruger, Marc Tennant

**Affiliations:** 1 Ministry of Health, Prince Nayef Street, Northern Abhor, Jeddah 23816, Saudi Arabia; dr-khalid-4108@hotmail.com; 2 International Research Collaborative- Oral Health and Equity, The University of Western Australia, Nedlands 6009, Australia; marc.tennant@uwa.edu.au

**Keywords:** primary health care, Ehalati, documented appointment scheduling rates, e-referrals, Saudi Arabia

## Abstract

Health systems are becoming more complex, regulatory bodies are increasing their vigilance, and reimbursement practices are shifting toward value, making closing the referral loop an imperative for patient safety, regulatory oversight, and financial viability. The aim of this study was to examine the referral pattern in PHC services and whether a significant variation exists among them based on geographic accessibility to a referred hospital. This was a cross-sectional retrospective study that included all sequentially referred patients between 1 January 2019 and 30 December 2021. A pre-initiative comparison could not be performed, as previous data on the traditional referral system could not be collected. The primary outcome measures considered in this study were the referral rate, and the proportion of the documented appointment date. The healthcare facilities’ geographic locations and data of the hospital departments to which the patients were referred were also available. Between 2019 and 2021, the hospital received 52,143 referrals from the 9 designated PHC centres covering 34 districts. In the PHC centres located within the ≤13 km zone, 1 in every 14 patients were referred to the hospital, whereas 1 in every 20 patients visited PHC centres outside this zone. Since the introduction of the Ehalati e-referral system, the number of documented appointment schedules of the referred patients has improved over time by 16.1% (from 79.6% to 95.7%, *p* < 0.001). Ophthalmologic (17.1%) and dental services (15.4%) received the most referrals among all other specialties, whereas the referral rate for cardiology services was the lowest (2.5%). The documented appointment scheduling record of referred patients has improved significantly since the introduction of the Ehalati e-referral system. However, the results of this study indicate that the proximity of PHC centres to specialised hospitals is more likely associated with higher referral and documented appointment scheduling rates. Strategies that improve scheduling, decrease variation among clinics, and improve patient access will likely improve the closing rates of the referral loop.

## 1. Introduction

Many healthcare systems place a high priority on the primary–secondary care interface. Primary care physicians provide primary health care (PHC) and determine which patients require secondary health care. Thus, strengthening the PHC sector is critical to improving healthcare access and quality [1]. While efforts have been made to improve PHC care, implementing effective interventions has been challenging. Insufficient financial resources, lack of political engagement, and insufficient management of referral patterns have hindered success. It is known that universal health care depends on access to comprehensive, appropriate, timely, and quality health services, without financial burden. Previous works have highlighted problems in terms of global health security, which does not address primary health care functions, including curative services, patient management, and capacity for clinical surges (as shown by the COVID-19 pandemic), as well as the need for securely exchanging health data [2,3]. A key element of PHC is its referral system, which enables patients to access higher levels of care from, for example, secondary and tertiary hospitals. As for health, the World Health Organization states that ‘referral is a process in which a health worker at one level of the health system, having insufficient resources (drugs, equipment, skills) to manage a clinical condition, seeks the help of a better or differently resourced facility at the same or higher level to assist in, or take over the management of, the client’s case’ [4]. Thus, to fill the existing gaps in the health infrastructure, a sound referral system is essential. However, various causes of failure of traditional, paper-based referral systems have been identified in the literature [5,6]. These include an untrained and unmotivated workforce, insufficient infrastructure, non-compliance with guidelines on how to establish an effective referral system, and lack of accountability to control unnecessary referrals at each level [7]. Consequently, under-utilisation of necessary specialised care will likely result in increased risks of patient morbidity and mortality.

Health systems are becoming more complex, regulatory bodies are increasing their vigilance, and reimbursement practices are shifting toward value, making closing the referral loop an imperative for patient safety, regulatory oversight, and financial viability. A failure to close the referral loop can result in poor outcomes, dissatisfied patients, and dissatisfied referring physicians, which can lead PHCs to refer their patients to out-of-network specialists, resulting in lower referral volumes and revenue for the practice.

In response to these issues, the Saudi Ministry of Health (MoH) has introduced the Ehalati system, an e-referral system, in PHC centres to expand access to appropriate facilities [8]. Although the MoH has launched the Ehalati system in 2017, this system has only recently become widely adopted by primary healthcare providers. In e-referral, or electronic referral, an electronic platform is used to transfer patient data seamlessly from a primary to a secondary or tertiary treating physician [7]. The e-referral system may reduce the distance between periphery and tertiary care centres, which will enable health workers to manage cases in a timely manner, will enhance the quality of continuity of care for patients, and will streamline the current unorganised referral process. Despite these advantages, the new system has not yet been fully examined for its impact. However, a closer review of the literature on PHC referral services in Saudi Arabia revealed that key information about the main referral factors for patients was often poorly reported [9]. Hence, the aim of this study was to examine the referral pattern in PHC services and whether a significant variation exists among them based on geographic accessibility to a referred hospital. In light of the complexity of referral tracking and its relatively recent scrutiny, we assume this analysis will highlight the challenges facing other health systems and provide insights into the root causes and areas for improvement.

## 2. Methods

### 2.1. E-Referral System

The King Abdullah Medical Complex (KAMC) in Northern Jeddah, Saudi Arabia, is a general hospital affiliated with the MoH. The hospital (with a capacity of 500 beds) provides diagnostic, medical examination, and therapeutic services. The hospital receives referrals from 9 designated PHC centres that cover 34 districts.

**Figure 1:**
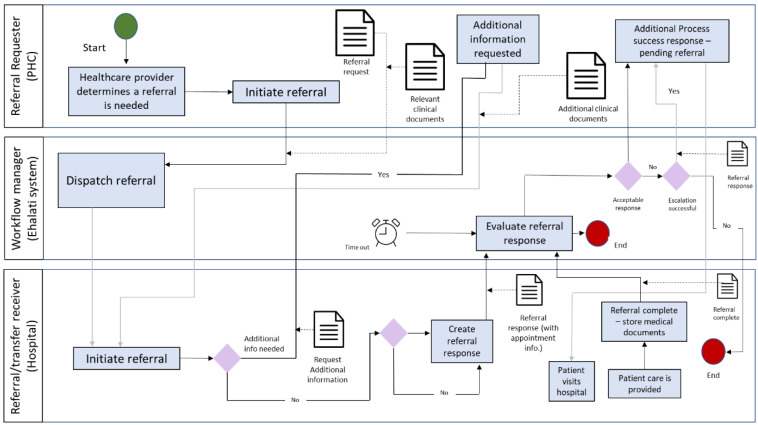
E-referrals process workflow [11].

Referrals are submitted electronically through the Ehalati system to the eligibility department at the KAMC. The Ehalati program is a national project that links all PHC centres of the MoH to an assigned tertiary hospital, with a view to expedite workflow (Figure 1). The eligibility department coordinates with the medical department and assigns the patient to the appropriate specialists. After that, appointments are scheduled for the patient on the basis of their case status. SMS (Short Message Service) messages informing on the appointments will then be sent to the patient. The SMS service for patient appointment is fully integrated with the appointment scheduling calendar software in the hospital. The MoH has provided a mobile application, Mawid, which allows beneficiaries to manage their referral appointments through a central appointment system [10].

### 2.2. Study Area

Jeddah is located on the west coastline of Saudi Arabia and has an area of approximately 1765 km^2^. The city has also expanded its services over the years to include public utilities such as water, electricity, and other infrastructures, as well as transportation, communication, and health-care projects. In terms of demographics, approximately 4.1 million people live in Jeddah (2015), making it Saudi Arabia’s second-largest city after its capital, Riyadh. The average household size is 5.2, and 41% of the population are younger than 23 years, whereas 3% are older than 65 years. The healthcare industry in Jeddah city comprises three main sectors: the MoH network of hospitals and PHC centres dispersed throughout the city, along with other governmental institutions and the private sector. This paper focuses only on PHC centres operated by the MoH.

### 2.3. Study Design, Setting, and Sample Selection

This was a cross-sectional retrospective study that included all patients referred from a selected PHC centre of the KAMC between 2019 and 2021. The PHC centre joined the MoH e-referral programme in January 2018. The study included all sequentially referred patients between 1 January 2019 and 30 December 2021. A pre-initiative comparison could not be performed, as previous data on the traditional referral system could not be collected.

### 2.4. Measures

This dataset included all referral orders made by primary care physicians and the resulting specialty appointment scheduling attempts. Associated data fields included the referral date, referral specialty, primary care clinic of origin, specialist appointment date, and urgency of the referral. The primary outcome measures considered in this study were the referral rate, which is the number of referrals divided by the number of consultations, and the proportion of the documented appointment date, which is the number of scheduling attempts divided by the total number of referred patients.

The relationship between the numbers of PHC visits and referrals was assessed as a ratio. Participants who visited the PHC centre during the study period were included. In this study, a PHC centre visit was defined as a face-to-face encounter between a patient and a physician, a nurse, or other PHC providers.

A map of Jeddah was also obtained from the General Authority of Statistics website. The geographic locations of the PHC centres and KAMC were also obtained from the General Administration of the MoH in the Jeddah region. PHC centre locations were geocoded using a free-access geocoding website for Google Maps, by which geographic coordinates (longitudes and latitudes) were assigned to the physical addresses of the locations. All the data collected were transferred into the Quantum-GIS software (version 2.14.1, QGIS Development Team (2021), QGIS Geographic Information System, Open-Source Geospatial Foundation Project) for analysis. The non-spatial data linked to those PHC centres included the number of medical referrals. To assess geographic accessibility, a buffer was created to quantify patient referrals in catchment areas. Buffers with a radius of 13 km were made around the KAMC (as an in-network referral hospital) and used as proxies for accessibility distances [12]. The PHC centres (as sites of referral) were grouped into accessible (within the 13 km buffer) and inaccessible (outside the 13 km buffer), and scheduled appointments were compared between the two groups.

Data of the hospital departments to which the patients were referred were also available. From the 52,143 scheduling attempts, we focused on 11 high-volume (>1000 referrals) medical and surgical specialties. The growing volume and complexity of care coordination networks, changing reimbursement models, and accrediting bodies exert more pressure; therefore, closing the referral loop is becoming crucial to the success of health care systems today, and for future health care systems as well. Additionally, in fee-for-service models, revenue is driven by volume of referrals to in-network specialists, which is influenced by patient outcomes, patient satisfaction, and referring provider satisfaction. Demographic data (e.g., age, sex, and nationality) were collected as the principal descriptive component of the study. All data identifying beneficiaries, physicians, and institutions were encrypted to ensure privacy.

### 2.5. Ethics Approval

Before conducting the research, an ethics application was submitted to the MoH of Saudi Arabia (KSA:H-02-J-002) and the Human Research Ethics Committee of the University of Western Australia (RA/4/20/6317). The research proposal was reviewed and approved. The data integrity and privacy were ensured by all means: participants in the extracted data were assigned numeric identifier, the database was password protected in the department computer, and access was restricted to selected investigators with completed confidentiality agreement. 

### 2.6. Statistical Analysis

For analysing the data, Statistical Package for Social Sciences version 21 (SPSS Inc., Chicago, IL, USA) was used. The data were first screened for any entry errors and outliers. Descriptive statistics were undertaken and categorical data (e.g., sex and number of referrals) were reported as frequency, percentage, and continuous variables (e.g., age as a mean and standard deviation). For inferential analysis, bivariate analyses using a chi-square test and *t* tests were performed to determine any statistically significant (*p* < 0.05) association between the explanatory categorical and continuous variables, with the distance from a referred hospital within the ≤13 or >13 km zone as the dependent variable.

## 3. Results

### 3.1. Practice Characteristics

**Figure 2:**
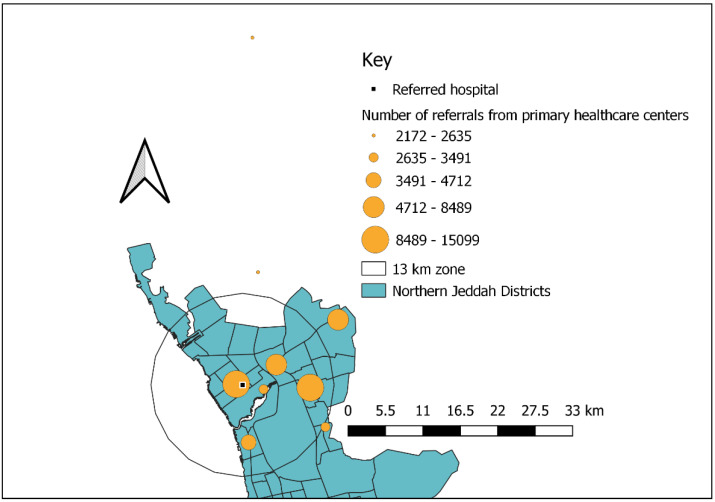
Distribution of referrals based on the primary healthcare service’ distance from a referred hospital between 2019 and 2021 (*n* = 52,143).

Between 2019 and 2021, the hospital received 52,143 referrals from the 9 designated PHC centres covering 34 districts. Only five of the PHC centres were located within the accessible zone (≤13 km; Figure 1). In the PHC centres located within the ≤13 km zone, 1 in every 14 patients were referred to the hospital, whereas 1 in every 20 patients visited PHC centres outside this zone (Table 1). Referrals varied considerably between the PHC centres within the accessible distance of the hospital (76.8%) and those within an inaccessible distance (23.2%; Figure 2).

### 3.2. Referral Characteristics

**Figure 3:**
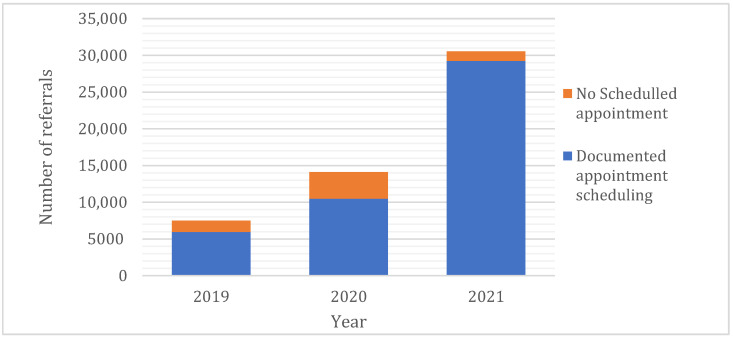
Distribution of referrals outcomes overtime (*n* = 52,143, 2019–2021).

Of the total number of referrals, 45,697 (87.6%) resulted in documented complete appointments (Table 1), of which 6446 had no documented appointment dates. However, stratification by primary care site of referral origin revealed a significant variation in documented appointment scheduling rates (14% ‘>13 km zone’ vs. 11.8% ‘≤13 km zone’; *p* < 0.001). Since the introduction of the Ehalati e-referral system, the number of documented appointment schedules of the referred patients has improved over time by 16.1% (from 79.6% to 95.7%, *p* < 0.001; Figure 3). Most referrals (80%) were categorised as non-urgent (Table 1).

### 3.3. Patients’ Characteristics

**Figure 4:**
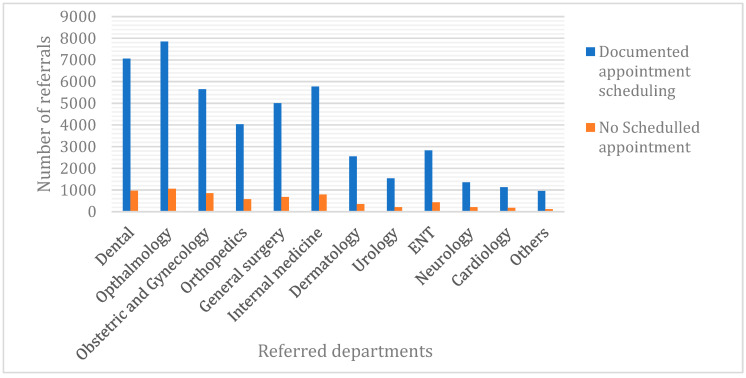
Distribution of referrals outcomes per department between 2019 and 2021 (*n* = 52,143).

Patients referred by PHC centres situated far from the hospital have a significantly higher likelihood of being younger (mean ± SD, 37.3 ± 17.8 years) than those referred by PHC centres near the hospital (41.5 ± 18.4 years, *p* < 0.001). At the level of the primary care site, no significant difference in referral rate was found between the male and female patients. Ophthalmologic (17.1%) and dental services (15.4%) received the most referrals among all other specialties (Figure 4), whereas the referral rate for cardiology services was the lowest (2.5%; Figure 4).

## 4. Discussion

In this cross-sectional retrospective study, the variation of the referral patterns in PHC services and the accessibility of a referred hospital were investigated. However, a review of the literature on PHC referral services in Saudi Arabia revealed that key information about the main referral factors is rarely reported. Our analysis revealed that shorter distances between PHC services and in-network hospital care services were more likely to be associated with higher medical referral rates, which indicates that the availability of specialist care affects referral rates. In spite of the small magnitude of this difference at roughly half a mile, the significant difference shows the smaller but still tangible impact of geographic access on documented appointment scheduling rates. Our results are similar to those reported in previous studies that showed that, generally, the opening of a district general hospital increased the number of referrals for those specialties now providing local consulting services [13]. In addition, this study shows that shorter distances impact the percentage of successful appointment scheduling and the number of referrals per PHC centre visit. Thus, distance can hinder patients with transportation barriers. Moreover, if in-network care is not accessible, patients may opt for out-of-network care, may not schedule an appointment, or may fail to show up for their appointments. Therefore, the impact of geography on closing the referral loop must be further examined by performing a detailed geospatial analysis.

This study shows that patients referred by PHC centres outside the 13 km radius tend to be younger than those referred by PHC centres closer to the hospital, and this result is both statistically and practically significant. One explanation for this is that the incidence rates of chronic diseases, disability, and age-adjusted mortality among young people who live far from full-service hospitals are higher than those among people who live close to these hospitals [14].

Another important finding is that referrals to ophthalmologic and dental services were the most prevalent among all specialties. In fact, some dental and ophthalmologic specialties are often available from hospitals and are mainly provided by specialised health professionals. However, the literature suggests that some patients are improperly referred, consuming health-care resources that could have been used elsewhere, and that some patients are inappropriately treated in primary care settings when they should have received specialist care [15,16,17,18,19]. The costs associated with inappropriate referrals to secondary care are unnecessary, notwithstanding the effects on waiting lists. Therefore, it is imperative that only patients with appropriate referrals are referred for secondary care. However, the extent to which the variation in referral rates is related to specialist supply is still unknown. Conversely, in our study, referrals for cardiology were lower than those for any other specialty. An explanation for this is that cardiovascular diseases are urgent in nature, and this clearly drives patients’ decision to seek hospital care immediately.

The documented appointment scheduling record of referred patients has improved significantly since the introduction of the Ehalati e-referral system. Several challenges could explain the small proportion of documented appointment schedules observed in 2019. A previous study in Riyadh (2014) reported that the implementation of Ehalati was hampered by the lack of computer skills and interest in the organisation’s new software among staff, and the lack of a clear change management strategy [6]. Perhaps this resulted from insufficient training and poor management of the staff. Among the reported *challenges* is the lack of appropriate technology, including the network, hardware, and workstations. At times, this might hinder the implementation of the referral system.

## 5. Limitations

The analyses of the patient referrals were limited to a single in-network hospital and the study is neither longitudinal nor experimental, which limits the extent to which a causal relation can be drawn and generalised. However, many of the challenges discussed above are generalizable to large health systems with complex referral networks. Additionally, our proxy measurement of wait time as days between referral order and appointment date is limited, as some patients choose to schedule later appointment dates than those available. Additionally, our proxy measurement of geographic access is limited, as true geographic access measurement would be distance between each patient’s address and the hospital assigned to the appointment. As we had no data on patient addresses, we applied the proxy measures of PHC address and in-network hospital location only to scheduled appointments (nearly exclusively in-network referrals). Moreover, the claims data did not provide detailed demographic and socio-economic data, or medical backgrounds of the referred patients, which precluded analysis of possible contributing factors such as education level, insurance status and economic background. Similarly, as these patients had been examined by a general practitioner, this study was not able to assess the diagnostic accuracy of referrals. Within the context of this study, referral quality was therefore limited to assessing the medical department to which patients are of referred to. Consequently, referrals that are fully completed can still incur inappropriate patient triage if the content of the referral, especially the diagnosis, is insufficient, inaccurate, or incorrect. Lastly, this study fails to determine whether or not the patient attended the scheduled appointment; therefore, measuring the rate of closing the referral loop was not possible.

## 6. Implications and Recommendations

This study suggests the need to strengthen PHC and identify challenges in closing the referral loop, which is critical to ensure patient safety, as the referral process is a point of patient vulnerability. Failure to close the referral loop can lead to under-utilisation of necessary specialty care, increasing patient morbidity. The importance of closing the referral loop in health systems is increasing. Strategies that improve scheduling, decrease variation among clinics, and improve patient access will likely improve the closing rates of the referral loop. In addition, policymakers should pay attention to the variations in referral rates between PHC practices and between individual general practitioners, as they are perceived to have financial implications.

## 7. Conclusions

The documented appointment scheduling record of referred patients has improved significantly since the introduction of the Ehalati e-referral system. However, the results of this study indicate that the proximity of PHC centres to specialised hospitals is more likely associated with higher referral and documented appointment scheduling rates. Patients referred by PHC centres far from a specialised hospital have a significantly higher likelihood of being younger than those referred by PHC centres near a specialised hospital. PHC-wide variations in e-referrals to hospitals have implications for policymaking and funding interventions. Identified areas of weakness require action to improve referral rates and the usefulness of Ehalati systems.

## Figures and Tables

**Table 1:** Demographic and referrals characteristics based on the primary healthcare service’ distance from a referred hospital between 2019 and 2021 (*n* = 52,143).

Demographic and Referrals Characteristics		Total N(%)	Distance of the Referred Hospital N (%) or m ± SD ^a^		*p*-Value
			≤13 km Zone	>13 km Zone	
No. of Primary healthcare centres		9 (100)	5(55.6)	4 (44.4)	-
PHC visits to referrals ratio		16:1	14.5:1	20.4:1	-
Number of referrals		52,143 (100)	40,060 (76.8)	12,083 (23.2)	-
Documented scheduled appointment	Yes	45,697 (87.6)	35,305 (67.7)	10,392 (20)	0.001 *
	No	6446 (12.4)	4755 (9)	1691 (3.2)	
Referral’s urgency	Urgent	10,384 (20)	7237 (14)	3147 (6)	0.001 *
	Non-urgent	41,759 (80)	32,823(63)	8936 (17)	
Patient’s age (years) ^b^		40.5 ± 18.4	41.5 ± 18.4	37.3 ± 17.8	0.001 *
Gender	Males	22,869 (44)	17,553 (33.7)	5316 (10.2)	0.728
	Females	29,273 (56)	22,507 (43.2)	6767 (13)	

^a^ Standard deviation (SD), **^b^** *t*-test used for comparisons, * *p* < 0.05, deemed significant.

## Data Availability

The datasets analysed during this study are available from the corresponding author upon reasonable request.
